# The Effects of Different Doses of Canthaxanthin in the Diet of Laying Hens on Egg Quality, Physical Characteristics, Metabolic Mechanism, and Offspring Health

**DOI:** 10.3390/ijms25137154

**Published:** 2024-06-28

**Authors:** Junnan Zhang, Zhiqiong Mao, Jiangxia Zheng, Congjiao Sun, Guiyun Xu

**Affiliations:** State Key Laboratory of Animal Biotech Breeding and Frontier Science Center for Molecular Design Breeding, National Engineering Laboratory for Animal Breeding and Key Laboratory of Animal Genetics, Breeding and Reproduction, Ministry of Agriculture and Rural Affairs, Department of Animal Genetics and Breeding, College of Animal Science and Technology, China Agricultural University, Beijing 100193, China; cauzhangjn@163.com (J.Z.); zhiqiong_mao@163.com (Z.M.); jxzheng@cau.edu.cn (J.Z.); cjsun@cau.edu.cn (C.S.)

**Keywords:** canthaxanthin, production performance, egg quality, offspring physical characteristic, metabolic mechanism

## Abstract

Currently, there is a dearth of in-depth analysis and research on the impact of canthaxanthin on the production performance, egg quality, physical characteristics, and offspring health of laying hens. Furthermore, the metabolic mechanism of cantharidin in the body remains unclear. Therefore, to solve the above issues in detail, our study was conducted with a control group (C group), a low-dose canthaxanthin group (L group), and a high-dose canthaxanthin group (H group), each fed for a period of 40 days. Production performance was monitored during the experiment, in which L and H groups showed a significant increase in ADFI. Eggs were collected for quality analysis, revealing no significant differences in qualities except for yolk color (YC). The YC of the C group almost did not change, ranging from 6.08 to 6.20; however, the trend in YC change in other groups showed an initial intense increase, followed by a decrease, and eventually reached dynamic equilibrium. By detecting the content of canthaxanthin in the yolk, the YC change trend was found to be correlated with canthaxanthin levels in the yolk. The content of unsaturated fatty acid increased slightly in L and H groups. Following the incubation period, the physical characteristics and blood biochemical indices of chicks were evaluated. It was observed that the shank color of chicks in the L and H groups was significantly higher than that in the C group at birth. However, by the 35th day, there were no significant differences in shank color among the three groups. Further investigation into the metabolic mechanism involving canthaxanthin revealed that the substance underwent incomplete metabolism upon entering the body, resulting in its accumulation as well as metabolic by-product accumulation in the yolk. In summary, this study highlighted the importance of understanding canthaxanthin’s role in production performance, egg quality, and offspring health, providing valuable insights for breeders to optimize feeding strategies.

## 1. Introduction

Since the beginning of the 21st century, there has been a notable improvement in people’s living standards, leading to a growing emphasis on the significance of ‘nutritional food and a healthy diet’. Meat, eggs, and milk have emerged as cost-effective and nutritious options for livestock and poultry products, making them key components of people’s daily diets [1]. The widespread use of antibiotics and feed additives in the past has raised concerns about food safety, posing risks to human health, having potential ecological impacts, and hindering the sustainable growth of the livestock industry [2]. The European Union (EU) has permitted the addition of antibiotics to feed since the 1950s [3]. However, concerns regarding food safety have arisen. In 2006, the EU implemented a ban on the addition of antibiotics to feed [4], followed by China in 2020 according to the No. 194 announcement of the Ministry of Agriculture and Rural Areas. Consequently, the safe utilization of feed additives has become a hot topic in recent years. The ‘China Food Safety Development Report’ also highlights that the excessive use of feed additives contributes to the important challenges faced in food safety development [5].

Eggs, being a high-quality livestock and poultry product, are favored by consumers for their rich nutritional value. Preferences for egg consumption vary across regions, with some regions favoring eggs with darker yolks due to the belief that darker yolks indicate higher nutritional content and better flavor [6]. These colorants are added to poultry feed to improve the visual appeal and market value of the eggs. While natural colorants offer safety benefits, they are susceptible to various factors like environmental conditions and costs, leading to differences in stability and coloring effects [7]. In contrast, artificially synthesized colorants, such as carophyll red, are favored for their affordability, superior settling rate, and stability [8]. In carophyll red, which is a synthetic chemical colorant, canthaxanthin is the main active substance, belonging to the ketocarotenoid group with the chemical name 4,4-diketo-β-carotene and molecular formula C_40_H_52_O_2_.

Studies have shown that canthaxanthin, although altering the color of animal organisms and pigment deposition in products, does not provide any nutritional benefits [9]. This fat-soluble compound has the ability to accumulate in human adipose tissue, although it is primarily concentrated in the liver within bodies [10,11]. Additionally, it has been indicated that long-term consumption of canthaxanthin may impact human skin color and could potentially cause retinal lesions [12,13]. Therefore, the effect of canthaxanthin on poultry is always a question of primary interest.

**Figure 1:**
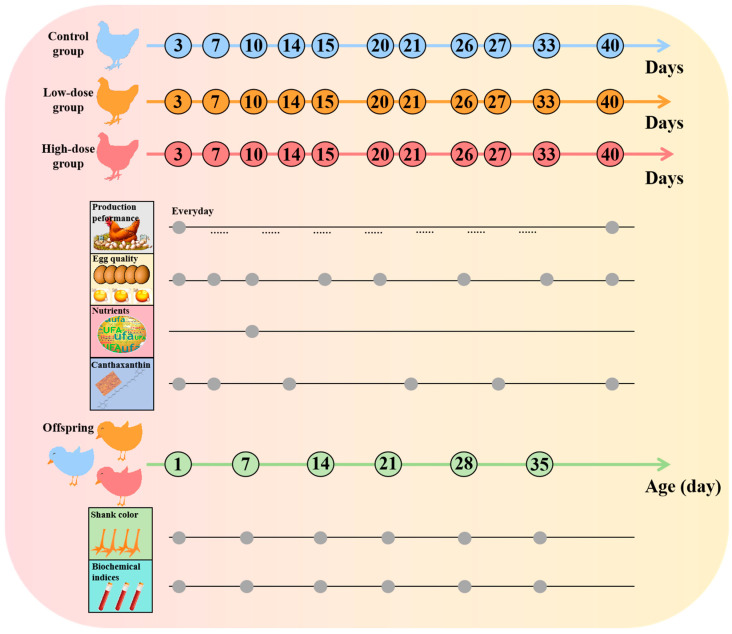
Study workflow of the present study.

Current research on incorporating canthaxanthin into feed primarily concentrates on production performance and egg quality. Canthaxanthin effectively improves yolk color, and canthaxanthin deposition in the yolk enhances the antioxidant function of laying hens [14,15]. Additionally, canthaxanthin can improve both productive and reproductive performance, enhancing egg production, egg quality, and hatchability [16]. However, there is a lack of more comprehensive research on reproductive performance, offspring health, and in vivo metabolic mechanisms. This study aimed to investigate the potential impacts of canthaxanthin, within the maximum allowable range (0–8 mg/kg) specified for poultry feed (according to the Chinese Agricultural Department Announcement No. 2625), on production performance, egg quality, reproductive capacity, offspring health, and metabolic mechanisms within the chicken body (Figure 1). The objective was to comprehensively assess the impact of canthaxanthin on poultry production and offspring, providing a theoretical foundation for the safe use of poultry feed additives.

## 2. Results

### 2.1. Production Performance 

As expected, canthaxanthin in the feed did not significantly affect most production performance measures of the three groups (Table 1), including the laying rate (LR), soft or broken egg rate (SBR), and feed conversion ratio (FCR, feed/egg). However, the average daily feed intake (ADFI) in the L and H groups was significantly higher than in the C group (*p* < 0.05), with no significant difference between the L and H groups (*p* > 0.05).

### 2.2. Egg Quality 

The egg qualities, including eggshell index (ESI), egg weight (EW), eggshell strength (ESS), eggshell thickness (EST), albumen height (AH), Haugh unit (HU), yolk color (YC), and yolk weight (YW), were analyzed across various groups at seven different time points. It was observed that canthaxanthin did not have a significant impact on ESI, ESS, EST, AH, HU, EW, and YW. The ESI ranged from 1.290 to 1.296 (mean: 1.29, coefficient of variation (CV): 0.29%); ESS ranged from 3.02 g to 3.24 g (mean: 3.15 g, CV: 2.14%); AH ranged from 4.71 mm to 5.91 mm (mean: 5.51 mm, CV: 7.82%); HU ranged from 66.74 to 76.47 (mean: 73.10, CV: 5.06%); EW ranged from 56.12 g to 58.52 g (mean: 57.57 g, CV: 1.28%); YW ranged from 17.13 g to 17.66 g (mean: 17.33 g, CV: 1.09%); and EST (blunt end, equator, pointy end) varied from 0.36 mm to 0.37 mm (mean: 0.37 mm, CV: 0.50%), 0.36 mm to 0.37 mm (mean: 0.37 mm, CV: 0.59%) and 0.39 mm to 0.40 mm (mean: 0.39 mm, CV: 0.43%) at different time points (Supplementary Materials).

**Figure 2:**
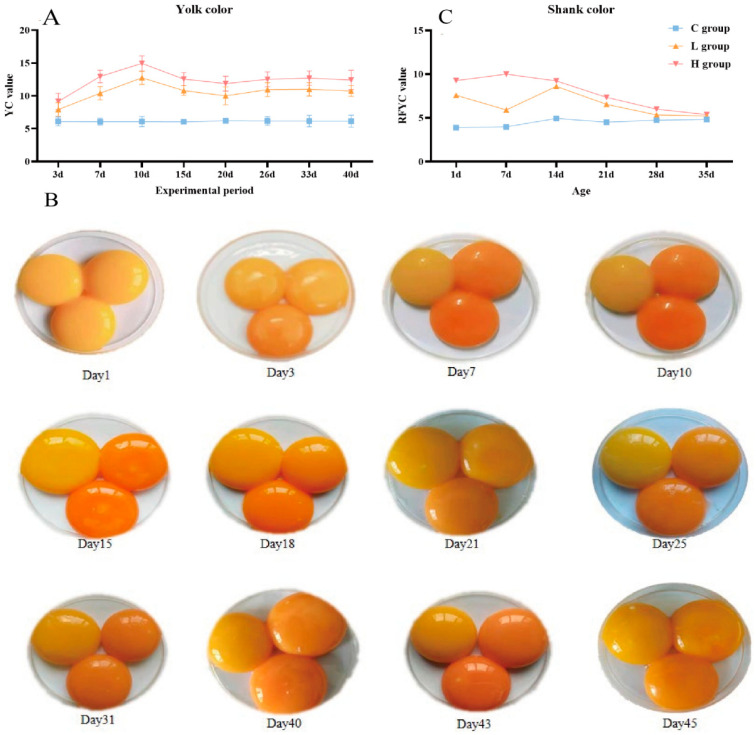
The changing trend of yolk color and shank color of offspring in three groups. (**A**,**B**) represent the RFYC value and visual changes. (**C**) represents the shank color of offspring, in each figure; the C group is shown in the upper left, the L group is in the upper right, and the H is in the lower part.

Compared with the C group, the YC was notably impacted by canthaxanthin in the feed, resulting in distinct variations across the three groups (H group > L group > C group). The C group consistently maintained a steady YC within the range of 6.08 to 6.20. In contrast, the L and H groups displayed a wider range of 7.92–12.76 and 9.16–14.95, respectively. Additionally, the peak value of YC was observed at 10 days, stabilizing between 15 and 40 days (Figure 2A,B).

### 2.3. Canthaxanthin and Nutrients of Yolk 

Our study revealed that canthaxanthin, when added to the feed of laying hens, was not completely metabolized in the body as it was eventually deposited into the yolk. Furthermore, an increase in the concentration of canthaxanthin in the feed resulted in higher deposition in the yolks. We observed that the canthaxanthin content in yolks initially increased and then decreased over the feeding period for both the L group and the H group. By the 14th day, the yolk’s canthaxanthin content peaked at values of 6.03 ± 0.29 mg/kg and 16.40 ± 2.1 mg/kg, respectively (Table 2). Lastly, when we analyzed the canthaxanthin content of egg yolks in the C group on the 3rd day, we did not find any canthaxanthin, leading to the decision to not test the C group again at subsequent time points.

The nutrients of the yolk were analyzed on the 10th day of the experimental period. Compared to the C group, the L group showed a decrease in vitamin D content but an increase in α-tocopherol and unsaturated fatty acids (UFAs). On the other hand, the H group exhibited an increase in vitamin D, α-tocopherol, and UFAs, with a decrease in the content of δ-tocopherol (Table 3).

### 2.4. The Offspring

#### 2.4.1. The Color of Physical Characteristic

**Figure 3:**
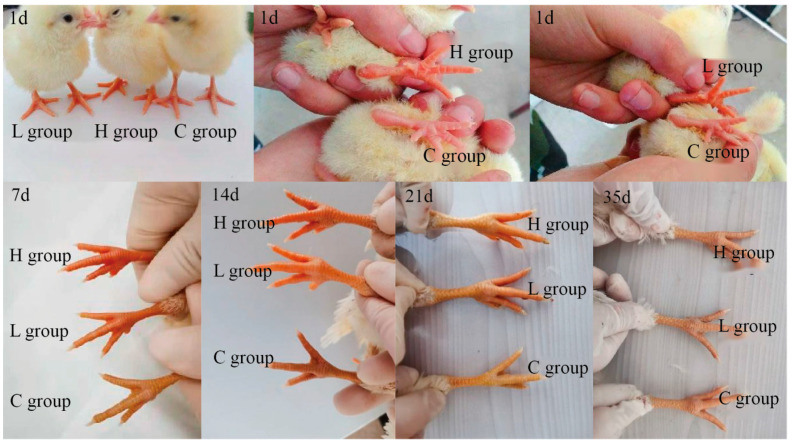
Dynamic changes in the shank color of offspring in the three groups.

The offspring’s physical characteristics, including feather and shank color, were investigated. The feather color had no differences among the various groups, while there were significant differences in shank color (Figure 2C and Figure 3). The C group exhibited a gradual darkening in shank color between 1 and 35 days post-birth, with the RYCF value ranging from 3.89 to 4.83. In contrast, the L and H groups displayed a gradual lightening in shank color during the same period, but their RYCF values were significantly higher than those in the C group. By the 35th day post-birth, the shank color of all groups appeared similar.

#### 2.4.2. The Biochemical Indices

**Figure 4:**
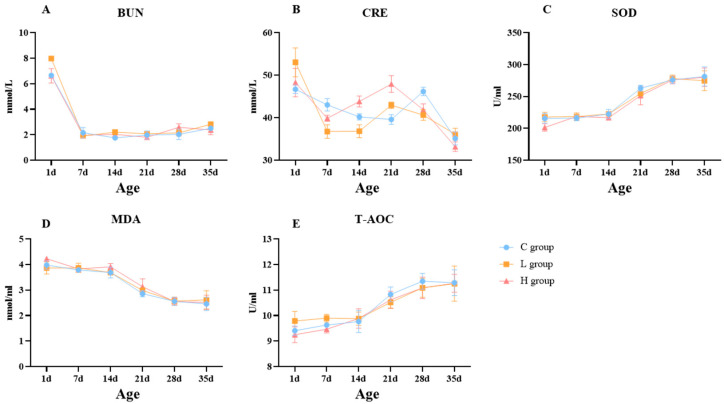
Dynamic changes in biochemical indexes (**A**–**E**). The blue line represents the C group, the orange line represents the L group, and the red line represents the H group.

To further investigate the impact of canthaxanthin on offspring, the level of creatinine (CRE), blood urea nitrogen (BUN), superoxide dismutase (SOD), total antioxidant capacity (T-AOC), and malondialdehyde (MDA) in serum were analyzed at 1d, 7d, 14d, 21d, 28d, and 35d (Figure 4). These five biochemical index trends were basically the same. The BUN level reached its peak at 1d and then decreased consistently across all groups. The CRE levels showed an initial decline, followed by an increase, and then another decline in all groups. However, the second turning point for the C group was at 28d, while for the L and H groups, it occurred at 21d. The SOD, MDA, and T-AOC levels were similar across all groups, with SOD and T-AOC levels increasing with age, while MDA levels decreased. Lastly, all these five blood biochemical indicators were observed with almost no difference among the three groups at 35d.

### 2.5. Metabolic Mechanism of Canthaxanthin in Organism

**Figure 5:**
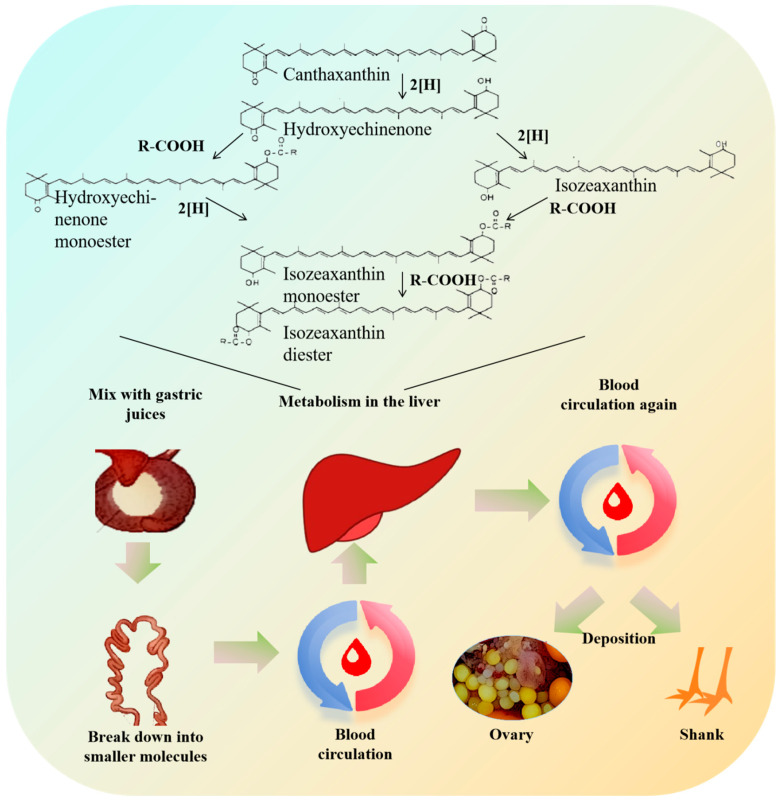
Metabolism of canthaxanthin in chicken.

Our study revealed that when chickens consumed canthaxanthin, the compound could be detected in the yolk, suggesting that canthaxanthin was not fully metabolized or excreted in the digestive system. Instead, it was transported to the ovary via blood circulation, thus influencing yolk formation, altering its color, and changing the shank color of offspring. Therefore, combined with the results of previous related studies [9,17,18], our results outline the digestive and metabolic processes of chicken post-canthaxanthin ingestion (Figure 5). Firstly, canthaxanthin enters the stomach through the esophagus, where pepsin aids in its decomposition. After being mixed with gastric juices, it moves to the intestines. In the intestines, canthaxanthin breaks down into smaller molecules, which are further broken down by intestinal digestive enzymes for absorption. Subsequently, canthaxanthin is transported through the blood to the liver. In the liver, a ketone group in canthaxanthin is reduced to an alcohol, forming hydroxyechinenone, which is then converted to isozeaxanthin. Some hydroxyechinenone reacts with isozeaxanthin, which produces hydroxyechinenone monoester, isozeaxanthin monoester, and isozeaxanthin diester. Ultimately, these metabolites are primarily concentrated in the shanks, leading to a change in shank color.

## 3. Discussion

In daily life, many individuals use the intensity of YC as a criterion for selecting eggs, with a common belief that darker YC signifies higher quality [19]. The YC is primarily influenced by the presence of pigments such as lutein and a small amount of carotene [20]. Consequently, some breeders utilize artificial methods, such as adding approved coloring agent canthaxanthin to the feed. The potential impact of canthaxanthin on production, egg quality, and offspring health has been a topic of ongoing concern. To address this issue, our study extensively examined the impact of canthaxanthin supplementation in the feed considering various aspects, including production performance, egg quality, reproductive capability, offspring characteristics and health, and canthaxanthin metabolism within the organism.

Treatment groups were established with different levels of canthaxanthin within the approved limits for poultry feed in accordance with national regulations. Our findings showed that the addition of canthaxanthin to the feed did not have a significant effect on LR, FCR, SBR, and FR. In contrast to previous studies [21,22], Rose et al. found no significant effects on SBR and FCR when 6 mg/kg of canthaxanthin was added to the feed for 42-week laying hens [23]. Grashorn also observed that while canthaxanthin from feed could transfer to egg yolk, it did not have a significant impact on production performance [24], which aligns with the results of our current study. However, in our study, we did observe a noticeable increase in ADFI in the presence of canthaxanthin in the feed. This could be attributed to the vivid color of canthaxanthin, which is easily assimilated into the feed, and its red hue enhances appetite, thereby boosting ADFI [25]. Furthermore, it was suggested that adding 6 mg/kg of canthaxanthin to the feed could enhance FR [26], which was consistent with our FR results.

Canthaxanthin primarily affected the YC quality parameter without significantly impacting EW, ESI, ESS, AH, HU, YW, and EST in this study. The increase in canthaxanthin dosage resulted in a darker YC, showing a dose-dependent response. Surai et al. demonstrated that adding 24 mg/kg of canthaxanthin to the feed changed YC from pale yellow to red without affecting other quality parameters [27]. On the 3rd day, the YC value was 7.92 in the L group and 9.16 in the H group, significantly higher than the C group. Visual observations supported the shift from yellow to red color. 

Canthaxanthin, a fat-soluble pigment, is easily absorbed and deposited in the body [28], with deposition efficiency in egg yolk ranging from 30% to 45% [29]. Our results demonstrated that the canthaxanthin content of yolks initially increased with feeding time, then decreased, and eventually reached a stable dynamic balance, implying that the early deposition of canthaxanthin in chicken is a rapid accumulation process. Higher canthaxanthin concentrations were detected on the 3rd day, with the L group containing 1.03 ± 0.01 mg/kg and the H group containing 1.68 ± 0.02 mg/kg, reaching maximum values on the 14th day of addition. Previous studies have also shown that the deposition of lutein (of which canthaxanthin is a type) into egg yolk is a rapid process [30,31], which aligns with our results. Additionally, canthaxanthin supplementation leads to an increase in vitamin D, α-tocopherol, and UFAs in the yolks, among which UFAs are known to be beneficial for human health [32] by lowering cholesterol, reducing the risk of cardiovascular disease, and providing antioxidant benefits [33,34]. Canthaxanthin’s ability to regulate free radicals in UFAs may have contributed to the increase in UFAs in yolks [35].

When hens consume the feed with canthaxanthin, a large amount of canthaxanthin is deposited in the ovary, which plays a role in the yolk deposition process. The yolk is an important source of nutrients for embryonic development [36]. Therefore, we sought to reveal the effect of canthaxanthin on the offspring through an incubation experiment. It was found that the shank color of offspring could be changed. The shank colors of 1-day offspring from the C, L, and H groups were measured at 3.9, 7.6, and 9.3, respectively. For 14-day chicks, there were also differences among the different groups, with shades of red observed in the L and H groups, which implies that canthaxanthin has a lasting influence on shin color. This trend continued until 35 days when all three groups exhibited yellow shanks. Variations in feather color were also noted among the offspring of the three groups, albeit without significant differences. Hence, considering the physical characteristics, canthaxanthin primarily changed the shank color. Finally, the changes in BUN, CRE, SOD, MDA, and T-AOC levels in serum were used to measure offspring health. BUN and CRE levels serve as indicators of renal function damage [37], while SOD, MDA, and T-AOC levels assess oxidative stress in the body [38]. Except for CRE, the overall trends in all indicators were consistent across the three groups, with no significant variations observed. Notably, CRE levels remained within an acceptable range of fluctuation, showing convergence across the three groups by day 35. This result suggests that incorporating canthaxanthin in poultry feed within a reasonable range is unlikely to have a negative impact on renal function and oxidative stress of offspring.

## 4. Materials and Methods

### 4.1. Animal Ethics

All experimental procedures in this study were reviewed and approved by the Animal Care and Use Committee of China Agricultural University (AW50213202-2-2). The experiment took place at the National Engineering Laboratory for Animal Breeding and the Key Laboratory of Animal Genetics, Breeding, and Reproduction, Ministry of Agriculture and Rural Affairs, China Agricultural University.

### 4.2. Experimental Design and Dietary Treatments

A total of 90 White Leghorn hens of 300 days with a similar body weight of 1653.12 ± 139.31 g/bird were selected from Beinongda Technology Co., Ltd. (Beijing, China), and randomly assigned to 3 dietary treatments, with 10 replicates per treatment and 3 hens per replicate. All the hens were housed individually in cages with an area of 500 cm^2^ under a light–dark cycle of 16 h light and 8 h dark (16L:8D), equipped with an individual feeder and water. Birds were allowed to adapt to experimental diets in the layer house for 7 days, and the formal experimental period lasted for 40 days. According to the assigned treatment groups, layers were fed with corn–soybean meals, and the control group (C group) was only fed the base diet. We chose carophyll red (DSM Nutritional Products, Ltd.) supplements in which the main active substance (10%) was canthaxanthin, and divided the groups into the high-dose group (H group, 8 mg/kg canthaxanthin) and the low-dose group (L group, 4 mg/kg canthaxanthin). The ingredients and nutrient composition of the experimental diets are shown in Table 4.

### 4.3. Production Performance and Egg Quality 

The performance of each laying hen was monitored on a daily basis in the formal experimental period (Supplementary Materials). The EN, LR, SBR, FCR, feed/egg, and ADFI were recorded and calculated.

LR (%) = (total egg number per day/total chicken number) × 100%;
SBR (%) = (total soft or broken egg number per day/total egg number per day) × 100%
FCR = ADFI/total EW per day.

The eggs were collected on specific days throughout the experimental period, and their qualities were assessed on the same day of collection (Table 5). The egg qualities that were evaluated included ESI, EW, ESS, EST, AH, HU, YC, and YW.

The modified ESI was employed according to previous research and calculated as ESI = egg length/egg width [39]. Eggs were weighed individually using a P601N electronic balance (Qinghai Corporation, Shanghai, China) with a digital readout to the precision of 0.01 g. The ESS was measured using a Model-III Eggshell Strength Tester (Robotmation, Tokyo, Japan). Eggshell membranes were removed, and EST was measured at three regions (pointy end, equator, and blunt end) using a digital caliper to the nearest 0.001 mm. The EMT-7300IIMultifunctional Egg Tester (Robotmation, Tokyo, Japan) facilitated the acquisition of AH, YC, and HU. The HU equation is as follows:

(1) $$HU=100log(H+7.57-{1.7W}^{0.37})$$

where H denotes AH (mm), and W represents EW (g) [13]. Yolks were separated from the contents of the eggs, and YW was also obtained using the P601N electronic balance (Qinghai Corporation, Shanghai, China). 

### 4.4. Artificial Insemination and Hatching Rate

Artificial insemination was conducted on the 3rd and 4th days of the experimental period at 3 pm (Table 5), with hatching eggs collected on subsequent days. The hatching eggs were then placed in an incubator following pre-warming at a temperature of 25–30 °C for 8 h. The standard operating temperature of the incubator was initially set at 38 °C, gradually decreasing by approximately 0.1 °C every 3 days. Humidity levels were maintained between 50 and 52%. Throughout the incubation period, the hatching eggs were automatically turned approximately every 2 H, with a tilting angle of about 45° for each turn. Following hatching, the fertilization rate (FR) was determined as follows:

FR (%) = (number of fertile eggs/number of eggs set) × 100

### 4.5. Determination of Canthaxanthin and Nutrients in Yolk

For canthaxanthin, 15 eggs were randomly selected from each group, and the yolks from 5 eggs in each group were combined into one sample for the content detection on the specific days (Table 5), using high-performance liquid chromatography (HPLC) following the SN/T 2327-2009 standard.

For the nutrients, on the 10th day of the formal experiment, a total of 6 eggs were collected from each group. Yolks from the different groups were combined to create a composite sample for the analysis of nutrient content, specifically focusing on vitamin D, vitamin E, and UFAs. 

The testing procedures were as follows: 

For the detection of vitamin D (according to GB 5009.82-2016, Method 4), the sample underwent saponification with a potassium hydroxide ethanol solution. In the case of starch-containing samples, enzymatic hydrolysis with amylase was carried out prior to extraction, purification, and concentration. The semi-prepared sample then underwent column chromatography separation using normal-phase high-performance liquid chromatography and reversed-phase high-performance liquid chromatography C18. Detection was performed using a UV or diode array detector, with quantification achieved through either the internal standard method or an external standard method. The detection of four isomers of vitamin E (δ-Tocopherol, γ-Tocopherol, α-Tocopherol, and β-Tocopherol) was conducted following the guidelines of GB 5009.82-2016, Method 1. After the saponification of the vitamin E sample and enzymatic hydrolysis of starch with amylase, the compound underwent extraction, purification, and concentration. Subsequent separation on a C30 or PFP reversed-phase liquid chromatography column and detection using a UV detector or fluorescence detector were carried out. For UFA detection (as per GB 5009.168-2016), fats were extracted with a hydrolysis–ether solution, followed by saponification and methylation under alkaline conditions to produce fatty acid methyl esters. Analysis using capillary column gas chromatography utilized the internal standard method for quantitatively determining the fatty acid methyl ester content. By taking into account the content and conversion coefficients of different fatty acid methyl esters, the fatty acid content was calculated.

### 4.6. The Impact on the Offspring

To further investigate the impact of canthaxanthin on offspring (60 chicks with each group), we utilized the Roche Yolk Color Fan (RYCF) to determine the shank color of chicks and analyzed serum biochemical indices after birth at different ages (1, 7, 14, 21, 28, and 35 days) for health assessment. The final RYCF value was determined as the average shank color recorded by 3 different workers. Serum biochemical indices were measured as follows: 

After allowing the blood to stand for 2–3 h, it underwent centrifugation at 1800 r/min for 10 min in a high-speed centrifuge (Eppendorf, Hamburg, Germany). The supernatant was then subjected to a second centrifugation at 1300 r/min for 5 min. The resulting supernatant was transferred into 1.5 mL centrifuge tubes and stored at −20 degrees Celsius for subsequent biochemical parameter measurements. BUN, CRE, SOD, MDA, and T-AOC levels in the serum were quantified using a ZY KHB-1280 automatic biochemical instrument (KHB, Shanghai, China).

### 4.7. Data Analysis

The experimental data were organized and calculated using Excel 2019. A one-way analysis of variance (ANOVA) was conducted using SPSS 23 software, and in the presence of significant differences, Duncan’s multiple comparisons were performed. The data are presented as “mean ± standard deviation”, where *p* values < 0.05 indicate significant differences [14]. Lowercase letters with the same superscript denote significant differences, while *p* values > 0.05 indicate non-significant differences; data with the same letter are considered similar. All figures were generated using GraphPad Prism 8 software.

## 5. Conclusions

Incorporating canthaxanthin into poultry feed could enhance ADFI and YC in laying hens while not significantly affecting other production performance measures and egg qualities. Canthaxanthin remained partially unmetabolized in the body and could be found in the yolk. The content of canthaxanthin initially increased, then decreased temporarily, and eventually reached a dynamic equilibrium. Finally, supplementing feed with canthaxanthin could enhance the shank color of offspring until 35 days without adverse effects on kidney function and antioxidant capabilities. In conclusion, these findings offer a potential scientific rationale for utilizing canthaxanthin in poultry feed and establish a crucial theoretical foundation for poultry production.

## Figures and Tables

**Table 1:** The production performance and reproduction capacity of the three groups ^1^.

Item ^2^	C Group	L Group	H Group
ADFI (g)	130.34 ± 1.94 ^b^	136.09 ± 2.38 ^a^	136.08 ± 2.35 ^a^
LR (%)	82.25 ± 7.26	83.59 ± 8.11	84.92 ± 5.94
FCR	2.28 ± 0.03	2.32 ± 0.06	2.33 ± 0.05
SBR (%)	1.13 ± 0.12	1.20 ± 0.03	1.15 ± 0.04
FR (%)	94.6	95.1	97.2

^1^ Each value is presented as mean ± standard deviation, except FR. In the same row, different letters in superscript indicate significant differences (*p* < 0.05). ^2^ ADFI: average daily feed intake, LR: laying rate, FCR: feed conversion ratio, SBR: soft or broken egg rate, FR: fertilization rate.

**Table 2:** The content of canthaxanthin in the yolk of the three groups at different time points ^1^.

Time Point	C Group	L Group	H Group
3d	0	1.03 ± 0.01 ^b^	1.68 ± 0.02 ^a^
7d	—	3.67 ± 0.15 ^b^	6.76 ± 0.03 ^a^
14d	—	6.03 ± 0.29 ^b^	16.40 ± 2.10 ^a^
21d	—	3.28 ± 0.02 ^b^	9.02 ± 0.04 ^a^
27d	—	3.90 ± 0.01 ^b^	6.04 ± 0.20 ^a^
40d	—	1.73 ± 0.03 ^b^	5.36 ± 0.19 ^a^

^1^ Each value is presented as mean ± standard deviation. In the C group, we detected the content of canthaxanthin examined in the yolk on 3d; — indicates that no test was performed.

**Table 3:** The content of nutrients in the yolk of the three groups.

Item	C Group	L Group	H Group
Vitamin D (μg/100 g)	6.03	5.16	8.24
δ-Tocopherol (mg/100 g)	0.17	0.18	<0.12
γ-Tocopherol (mg/100 g)	<0.04	<0.04	<0.04
α-Tocopherol (mg/100 g)	7.58	8.94	10.5
β-Tocopherol (mg/100 g)	<0.04	<0.04	<0.04
Total tocopherol (mg/100 g)	7.79	9.16	10.65
UFA (mg/100 g)	19.2	20.5	20.7

**Table 4:** Composition of laying hens’ diets.

Items ^1^	Group ^2^		
	C Group	L Group	H Group
Canthaxanthin	-	4 mg/kg	8 mg/kg
Ingredient, %			
Corn	61.06		
Soybean meal	23.8		
Soybean oil	0.8		
Wheat bran	3		
Calcium hydrogen phosphate	0.9		
Small stone	9		
Sodium chloride	0.35		
Antioxidant	0.04		
Mold inhibitor	0.05		
Premix ^3^	0.5		
Maifan stone	0.5		
Total	100		
Nutrient composition, %			
AME, MJ/kg	11.13		
Protein	16.13		
Lysine	0.87		
Methionine	0.24		
Calcium	3.71		
Total phosphorus	0.58		

^1^ AME = apparent metabolizable energy. ^2^ C group = control group only fed the base diet, L group = the group using low-dose canthaxanthin supplements, H group = the group using high-dose canthaxanthin supplements. ^3^ Premix: mineral premix provided per kg of diet: Mn, 100 mg; Fe, 65 mg; Zn, 100 mg; Cu, 9 mg; I, 1 mg; Se, 0.3 mg. Vitamin premix provided per kg of diet: vitamin A, 10,000 International Units (IUs); vitamin D3, 3000 IU; vitamin E, 30 IU; vitamin K3, 4 mg; vitamin B1, 3 mg; vitamin B2, 8 mg; vitamin B6, 6 mg; vitamin B12, 0.03 mg; biotin, 0.3 mg; folic acid, 0.3 mg; pantothenic acid, 18 mg; niacin, 40 mg.

**Table 5:** The specific days of detecting egg quality and canthaxanthin and artificial insemination.

Day ^1^	Detect Egg Quality	Detect Canthaxanthin	Artificial Insemination
1			
3	√	√	√
4			√
5			
7	√	√	
10	√		
14		√	
15	√		
20	√		
21		√	
26	√		
27		√	
33	√		
40	√	√	

^1^ All the days were in the experimental period.

## Data Availability

The original contributions presented in the study are included in the article and Supplementary Material, further inquiries can be directed to the corresponding author.

## Supplementary Materials

The following supporting information can be downloaded at: https://www.mdpi.com/article/10.3390/ijms25137154/s1.
