# Supplementary material for: The Effects of Different Doses of Canthaxanthin in the Diet of Laying Hens on Egg Quality, Physical Characteristics, Metabolic Mechanism, and Offspring Health
Source: Int J Mol Sci. 2024 Jun 28;25(13):7154. doi: 10.3390/ijms25137154 (PMC11241014; doi:10.3390/ijms25137154)
Supplement: Supplementary file 1 [file ijms-25-07154-s001.zip › ijms-3041183-supplementary.pdf]

Table S1. The ESI and ESS of three groups at different time points[1]

Time point	ESI			ESS		
	C group	L group	H group	C group	L group	H group
3d	1.29±0.05	1.28±0.06	1.31±0.06	3.06±0.65	3.03±0.85	2.98±0.91
7d	1.29±0.06	1.31±0.06	1.30±0.12	3.15±0.78	3.05±0.62	3.06±0.62
10d	1.30±0.04	1.28±0.19	1.29±0.06	3.09±0.91	3.23±0.91	3.19±0.66
15d	1.28±0.06	1.31±0.05	1.29±0.05	3.15±0.75	3.18±0.91	3.19±0.73
20d	1.29±0.05	1.29±0.07	1.29±0.06	3.20±0.23	3.23±0.72	3.13±0.96
26d	1.29±0.06	1.30±0.05	1.28±0.05	3.17±0.88	3.21±0.92	3.14±1.02
33d	1.31±0.04	1.29±0.06	1.27±0.05	3.25±0.43	3.29±0.94	3.18±0.91
40d	1.30±0.06	1.31±0.05	1.28±0.05	3.20±0.79	3.25±0.87	3.10±1.12

[1]ESI: eggshell index, ESS: eggshell strength.

Table S2. The AH and HU of three groups at different time points[1]

Time point	AH			HU		
	C group	L group	H group	C group	L group	H group
3d	5.84±1.91	5.71±1.20	5.90±2.19	75.5±12.20	75.08±12.0	78.7±18.18
7d	5.88±1.05	5.97±0.82	5.87±1.34	76.24±5.45	76.98±5.68	76.19±8.96
10d	5.79±0.91	5.82±0.83	5.76±1.03	75.87±6.12	75.93±5.86	75.13±7.62
15d	5.82±1.12	5.94±0.75	5.68±1.03	75.61±6.27	76.99±5.51	74.39±9.85
20d	5.65±1.20	5.63±1.47	5.68±1.01	73.19±8.89	73.46±10.8	74.70±6.86
26d	5.19±0.97	5.23±0.94	5.21±0.81	70.23±8.10	70.60±9.07	70.41±7.74
33d	5.16±1.35	5.19±1.46	5.14±1.18	70.04±13.15	70.85±17.4	67.98±10.9
40d	4.78±1.47	4.83±1.36	4.51±1.73	67.22±10.03	68.10±10.3	64.90±8.23

[1]AH: albumen height, HU: haugh unit.

Table S3. The YW and EW of three groups at different time points[1]

Time point	YW			EW		
	C group	L group	H group	C group	L group	H group
3d	17.43±1.55	17.50±1.63	16.78±1.63	57.08±3.54	57.58±4.18	56.88±3.82
7d	17.33±1.22	17.33±1.61	16.94±1.48	58.23±3.95	58.02±4.62	56.67±4.67
10d	17.18±1.45	17.72±1.54	17.41±1.80	57.69±4.12	57.72±3.84	57.93±4.84
15d	17.14±2.11	17.10±1.21	17.15±2.08	57.23±3.95	57.57±3.45	57.66±5.31
20d	17.95±1.23	17.81±1.40	17.22±1.37	56.56±4.89	58.01±4.24	57.82±4.17
26d	17.77±1.34	17.57±1.11	16.38±2.26	58.34±3.48	58.35±4.39	58.87±5.83
33d	17.17±1.64	17.31±1.51	17.16±1.39	57.10±5.45	56.12±7.23	61.80±4.49
40d	17.38±1.73	17.62±1.38	17.59±1.38	56.79±4.28	55.07±5.54	56.49±3.88

[1]YW: yolk weight, EW: egg weight.

Table S4. The EST of three groups at different time points[1]

Time point	C group			L group			H group		
	blunt	equator	pointy	blunt	equator	pointy	blunt	equator	pointy
3d	0.36±0.04	0.37±0.02	0.38±0.03	0.38±0.03	0.36±0.04	0.39±0.03	0.35±0.02	0.37±0.03	0.39±0.03
7d	0.37±0.03	0.37±0.03	0.39±0.04	0.38±0.02	0.35±0.04	0.40±0.04	0.36±0.02	0.35±0.04	0.40±0.03
10d	0.36±0.03	0.38±0.02	0.39±0.03	0.37±0.03	0.36±0.02	0.39±0.02	0.38±0.03	0.38±0.04	0.41±0.02
15d	0.35±0.02	0.38±0.04	0.40±0.03	0.36±0.04	0.37±0.02	0.39±0.03	0.37±0.02	0.37±0.04	0.38±0.04
20d	0.36±0.03	0.37±0.03	0.38±0.02	0.36±0.03	0.35±0.03	0.39±0.04	0.39±0.03	0.38±0.03	0.41±0.03
26d	0.37±0.02	0.38±0.02	0.39±0.02	0.38±0.03	0.37±0.02	0.41±0.03	0.38±0.03	0.37±0.02	0.39±0.03
33d	0.36±0.03	0.37±0.03	0.38±0.03	0.37±0.03	0.38±0.03	0.39±0.04	0.38±0.03	0.36±0.02	0.39±0.02
40d	0.36±0.03	0.38±0.02	0.39±0.03	0.36±0.03	0.35±0.04	0.38±0.02	0.38±0.02	0.36±0.03	0.40±0.03

[1]EST: eggshell thickness.

Table S5. Egg number and laying rate

Time point	Egg number			laying rate		
	C group	L group	H group	C group	L group	H group
1d	23	25	25	76.67%	83.33%	83.33%
2d	26	23	23	86.67%	76.67%	76.67%
3d	23	24	23	76.67%	80.00%	76.67%
4d	26	25	24	86.67%	83.33%	80.00%
5d	27	22	23	90.00%	73.33%	76.67%
6d	23	25	23	76.67%	83.33%	76.67%
7d	24	25	24	80.00%	83.33%	80.00%
8d	24	25	24	80.00%	83.33%	80.00%
9d	24	28	25	80.00%	93.33%	83.33%
10d	24	25	23	80.00%	83.33%	76.67%
11d	25	24	28	83.33%	80.00%	93.33%
12d	27	22	27	90.00%	73.33%	90.00%
13d	27	28	23	90.00%	93.33%	76.67%
14d	25	25	27	83.33%	83.33%	90.00%
15d	24	22	23	80.00%	73.33%	76.67%
16d	26	23	27	86.67%	76.67%	90.00%
17d	25	18	27	83.33%	60.00%	90.00%
18d	26	23	26	86.67%	76.67%	86.67%
19d	28	26	26	93.33%	86.67%	86.67%
20d	20	26	27	66.67%	86.67%	90.00%
21d	25	28	26	83.33%	93.33%	86.67%
22d	27	20	27	90.00%	66.67%	90.00%

23d	24	24	27	80.00%	80.00%	90.00%
24d	26	24	28	86.67%	80.00%	93.33%
25d	15	25	27	50.00%	83.33%	90.00%
26d	25	28	26	83.33%	93.33%	86.67%
27d	25	26	26	83.33%	86.67%	86.67%
28d	27	25	27	90.00%	83.33%	90.00%
29d	25	24	24	83.33%	80.00%	80.00%
30d	23	25	29	76.67%	83.33%	96.67%
31d	23	26	25	76.67%	86.67%	83.33%
32d	26	28	24	86.67%	93.33%	80.00%
33d	25	25	22	83.33%	83.33%	73.33%
34d	25	27	27	83.33%	90.00%	90.00%
35d	25	28	27	83.33%	93.33%	90.00%
36d	26	25	25	86.67%	83.33%	83.33%
37d	25	26	27	83.33%	86.67%	90.00%
38d	24	30	25	80.00%	100.00%	83.33%
39d	24	25	27	80.00%	83.33%	90.00%
40d	25	30	25	83.33%	100.00%	83.33%
